# Single Cell Protein—State-of-the-Art, Industrial Landscape and Patents 2001–2016

**DOI:** 10.3389/fmicb.2017.02009

**Published:** 2017-10-13

**Authors:** Anneli Ritala, Suvi T. Häkkinen, Mervi Toivari, Marilyn G. Wiebe

**Affiliations:** VTT Technical Research Centre of Finland Ltd., Espoo, Finland

**Keywords:** single cell protein, SCP, algae, bacteria, fungi, microbial protein, Quorn™

## Abstract

By 2050, the world would need to produce 1,250 million tonnes of meat and dairy per year to meet global demand for animal-derived protein at current consumption levels. However, growing demand for protein will not be met sustainably by increasing meat and dairy production because of the low efficiency of converting feed to meat and dairy products. New solutions are needed. Single cell protein (SCP), i.e., protein produced in microbial and algal cells, is an option with potential. Much of the recent interest in SCP has focused on the valorisation of side streams by using microorganisms to improve their protein content, which can then be used in animal feed. There is also increased use of mixed populations, rather than pure strains in the production of SCP. In addition, the use of methane as a carbon source for SCP is reaching commercial scales and more protein-rich products are being derived from algae for both food and feed. The following review addresses the latest developments in SCP production from various organisms, giving an overview of commercial exploitation, a review of recent advances in the patent landscape (2001–2016) and a list of industrial players in the SCP field.

## Introduction

Humans and animals consume protein as a source of nitrogen and essential amino acids, from which they build new structural and functional (e.g., enzymes and hormones) proteins that enable them to survive. In extreme conditions, proteins may also be used as a source of energy. The nutritional value of a protein is determined by the amino acid composition; 20 amino acids are commonly found in dietary protein, of which several (i.e., phenylalanine, valine, threonine, tryptophan, methionine, leucine, isoleucine, lysine, and histidine, with arginine, cysteine, glycine, glutamine, proline, and tyrosine also being beneficial) cannot be synthesised by humans or animals and are thus essential and have to be supplied through the diet (for a review see Wu, 2009).

Boland et al. (2013) have explored how an increasing demand for meat and dairy protein will require improvements in animal production, as well as openness to new sources of protein, both as animal feed and for direct human consumption. Animal and dairy production have been increasing steadily over recent decades and theoretically can continue to do so to meet the expected demand, even by 2050 when the demand for meat would surpass 400 M tonnes and that for dairy 800 M tonnes (Boland et al., 2013). However, because plant protein is converted rather inefficiently into meat protein (~6 kg of plant protein is needed to produce 1 kg of meat protein), increasing meat production to match the growing demand is ultimately not sustainable (WHO, 2015). The western world is also interested in developing healthier food, with optimal amino acid composition and low, but good quality fat, combined with ethically sustainable production. These are typically non-animal based, environmentally friendly processes, but may include novel processes such as the production of “cultured” meat, in which meat protein is more efficiently produced *in vitro* rather than by growing an entire animal (Kadim et al., 2015). Plant-based protein sources, such as beans, are nutritionally valuable sources of protein, but require arable land and water, both of which will become limiting as we strive to meet global protein demand. The protein content of meat is generally about 45%, while that of milk is about 25% and of soybean about 35% (Ghasemi et al., 2011).

Protein can also be provided through the cultivation of various microbes and algae, preferably those which contain more than 30% protein in their biomass and which can provide a healthy balance of essential amino acids. Microbial protein is generally referred to as single cell protein (SCP), although some of the producing microbes, such as filamentous fungi or filamentous algae, may be multicellular. In addition to direct use as SCP, microbes contribute to protein demand when they are used to upgrade the protein content or quality of fermented foods (Bourdichon et al., 2012). Although, microbial protein provides a relatively small proportion of current human nutrition, the growing global demand for protein is likely to make SCP increasingly important (Boland et al., 2013). High growth rates or ability to utilise unique substrates, such as CO_2_ or methane, result in processes which offer much higher efficiency and/or sustainability than is possible from traditional agriculture.

SCP is currently produced from a limited number of microbial species, particularly when considering human consumption. The range of sources for SCP used in animal feed is broader than that approved for human consumption and is expanding. As is reviewed below, products derived from algae, fungi (including yeast) and bacteria are all in use or under development. The production steps generally include (a) preparation of nutrient media, possibly from waste, (b) cultivation, including solid state fermentation, (c) separation and concentration of SCP, in some cases drying, and (d) final processing of SCP into ingredients and products.

SCP for human consumption is generally produced from food grade substrates, but there is hope that processes will be developed to produce SCP from inexpensive waste materials from the food and beverage processing industries, as well as directly from forestry and agricultural sources (Anbuselvi et al., 2014). Regulatory issues must always be taken into account. With the introduction of algae to microbial protein providers, production from CO_2_ has become possible, while the greenhouse gas methane is providing a novel source of carbon for SCP from bacteria.

The following review will give an introduction to SCP production and the organisms used as SCP, with a focus on commercially implemented developments in the field. More detailed reviews of research with specific organisms considered for SCP production are provided by Anupama and Ravindra (2000), Ugalde and Castrillo (2002), Rudravaram et al. (2009), Ghasemi et al. (2011), and Nasseri et al. (2011). Here we provide an update on recent advances in the patent landscape (2001–2016) and the current industrial players, based on company profiles found from the web, literature and patent databases.

## SCP production systems with different substrates and processes

Algae, fungi (filamentous fungi and yeast), and bacteria can all be used as SCP (Anupama and Ravindra, 2000). In the future, dietary protein may also be derived from proteins secreted by engineered microbial cells (e.g., milk or egg white proteins) and produced from animal and plant cell cultures, in which the cells are no longer microbes but are not animals or plants, either. Thus, the distinction of what is SCP and what is other protein becomes blurred.

### SCP from algae

Microalgae which are produced for human or animal consumption typically have high protein content (e.g., 60–70%; Table 1). They also provide fats (with ω-3 fatty acids and carotenoids being of particular interest), vitamins A, B, C, and E, mineral salts, and chlorophyll (Gouveia et al., 2008). They have relatively low nucleic acid content (3–8%; Nasseri et al., 2011).

**Table 1:** Recent reports of the protein content of some algae that are of interest as SCP^*^.

**Organism**	**Protein content (%)**	**References**
*Aphanizomenon flos-aquae*	60–75	https://bluegreenfoods.com/nutritional-analysis/
		http://klamathvalley.com/aphanizomenon-flos-aquae/
*Aphanothece microscopica*	42	Zepka et al., 2010
*Arthrospira maxima* (*Spirulina maxima*)	60–71	De Oliveira et al., 1999
*Arthospira platensis (Spirulina platensis)*	46–63	Rafiqul et al., 2005
*Chlorella pyrenoidosa*	45	Waghmare et al., 2016
*Chlorella sorokiana*	46–65	Safafar et al., 2016
*Chlorella* spp.	62–68	Liu et al., 2013
*Chlorella vulgaris*	42–55	Li et al., 2013; Safafar et al., 2016
*Euglena gracilis*	50–70	Rodríguez-Zavala et al., 2010
*Scenesdesmus obliquus*	30–50	Duong et al., 2015; Apandi et al., 2017

* *Cells are typically cultivated with CO_2_ (or bicarbonate) and light, but effluent waters are often used to provide additional carbon, as well as other nutrients*.

Microalgae are currently used mainly in the form of supplements, available in tablet, capsule or liquid form, but they are increasingly also processed as ingredients which can be included in pastas, baked goods, snacks, and so on (Gouveia et al., 2008; Zimberoff, 2017). The most accessible commercial products are derived primarily from *Arthrospira platensis* and *Arthrospira maxima* (sold as spirulina, marketed by e.g., Hainan Simai Pharmacy Co., Earthrise Nutritionals, Cyanotech Corp., FEBICO, and Mayanmar Spriulina Factory)*, Chlorella* (marketed by e.g., Taiwan Chlorella Manufacturing Co., FEBICO and Roquette Klötze GmbH & Co), *Dunaliella salina* (marketed by e.g., Qianqiu Biotechnology Co., Ltd., primarily for β-carotene) and *Aphanizomenon flos-aquae* (marketed by e.g., Blue Green Foods, Klamath Valley Botanicals LLC and E3Live; Gouveia et al., 2008). Euglena Co. Ltd. (Suzuki, 2017) and Algaeon (http://algaeon-inc.com/#products) are both selling products from *Euglena*, primarily for the β-glucan content, but including whole cell products. TerraVia does not specify the alga provided in their AlgaVia® food ingredient. Enzing et al. (2014) and Vigani et al. (2015) provide useful surveys of the companies and countries involved in production of microalgae as food or feed. Both reviews focus on the European Union, but take note of the involvement of numerous companies in Asia and North America in the industry.

Algae generally feed on CO_2_ and light, although some products such as AlgaVia® are produced by traditional fermentation rather than by photosynthesis. Outdoor production of algae in open ponds is common, but is subject to contamination (not only biological contamination, but also mineral contamination which affects the quality of the final product) and variation in the weather (Harun et al., 2010). Indoor photobioreactors are also being used to guarantee the supply of fresh algae as feed for aquaculture (molluscs, shrimp, fish; Henrikson, 2013; Mahmoud et al., 2016). Algae are primarily used in aquaculture as a source of omega fatty acids and carotenoid pigments, but their protein also contributes to animal nutrition (Muller-Feuga, 2000).

### SCP from fungi

A wide range of fungi have been considered for use as SCP, as reviewed by Anupama and Ravindra (2000), Rudravaram et al. (2009), and Nasseri et al. (2011). Table 2 lists some of the species that have been researched in recent years, with the protein content observed under the conditions in which they were grown. Products from *Saccharomyces, Fusarium*, and *Torulopsis* are commercially available.

**Table 2:** Recent reports of fungal protein content produced from specific substrates for species investigated as potential sources of SCP.

**Organism**	**Substrate**	**Protein content (%)**	**References**
*Aspergillus flavus*	Rice bran	10	Valentino et al., 2016
*Aspergillus niger*	Apple pomace	17-20	Bhalla and Joshi, 1994
	Banana wastes	18	Baldensperger et al., 1985
	Rice bran	11	Valentino et al., 2016
	Stickwater	49	Kam et al., 2012
	Potato starch processing waste	38	Liu et al., 2013, 2014
	Waste liquor	50	Chiou et al., 2001;
*Aspergillus ochraceus*	Rice bran	10	Valentino et al., 2016
*Aspergillus oryzae*	Rice bran (deoiled)	24	Ravinder et al., 2003
*Candida crusei*	Cheese whey	48	Yadav et al., 2014
*Candida tropicalis*	Molasses	56	Gao et al., 2012
	Bagasse	31	Pessoa et al., 1996
*Candida utilis*	Poultry litter; Waste capsicum powder	29 48	Zhao et al., 2010; Jalasutram et al., 2013
	Potato starch industry waste	46	Liu et al., 2013
*Chrysonilia sitophilia*	Lignin	39	Rodriguez et al., 1997
*Cladosporium cladosporioides*	Rice bran	10	Valentino et al., 2016
*Debaryomyces hansenii*	Brewery's spent grains hemicellulosic hydrolysate	32	Duarte et al., 2008
*Fusarium semitectum* and *sp1 and sp2*	Rice bran	10	Valentino et al., 2016
*Fusarium venenatum*	Glucose (Product:Quorn™)	44	Wiebe, 2002
*Hanseniaspora uvarum*	Spoiled date palm fruits	49	Hashem et al., 2014
*Kefir* sp.	Cheese whey	54	Paraskevopoulou et al., 2003
	Orange pulp, molasses, brewer's spent grain, whey, potato pulp, malt spent rootlets	24-39	Aggelopoulos et al., 2014
*Kluyveromyces marxianus*	Cheese whey	43	Yadav et al., 2014, 2016
	Orange pulp, molasses, brewer's spent grain, whey, potato pulp	59	Aggelopoulos et al., 2014
*Monascus ruber*	Rice bran	10	Valentino et al., 2016
Unspecified, marine yeast	Prawn shell wastes	61–70	Rhishipal and Philip, 1998
*Penicillium citrinum*	Rice bran	10	Valentino et al., 2016
*Pleurotus florida*	Wheat straw	63	Ahmadi et al., 2010
*Saccharomyces cerevisiae*	Orange pulp, molasses, brewer's spent grain	24	Aggelopoulos et al., 2014
*Trichoderma harzianum*	Cheese whey filtrate	34	Sisman et al., 2013
*Trichoderma virideae*	Citrus pulp	32	De Gregorio et al., 2002
*Yarrowia lipolytica*	Inulin, crude oil, glycerol waste hydrocarbons	48–54	Cui et al., 2011; Zinjarde, 2014

Fungi grown as SCP will generally contain 30–50% protein (Anupama and Ravindra, 2000; Nasseri et al., 2011). The amino acid composition compares favourably with the FAO guidelines; threonine and lysine content is typically high, but methionine content relatively low, although still meeting the FAO/WHO recommendations (Anderson et al., 1975). The methionine content of some fungal products such as Marmite® is even lower. Sulphur containing amino acids have been enriched in SCP from *K. fragilis* by cultivation on whey (Willetts and Ugalde, 1987).

In addition to protein, SCP derived from fungi is expected to provide vitamins primarily from the B-complex group (thiamine, riboflavin, biotin, niacin, pantothenic acid, pyridoxine, choline, streptogenin, glutathione, folic acid, and p-amino benzoic acid). The cell walls of fungi are rich in glucans, which contribute fibre to the diet. Low-density lipoprotein cholesterol has been reduced in volunteers who consumed myco-protein from *Fusarium venenatum* (Turnbull et al., 1992) and blood glucose and insulin levels may also be favourably affected (Lang et al., 1999). Fungi are expected to have a moderate nucleic acid content (7–10%; Nasseri et al., 2011), which however is too high for human consumption and requires processing to reduce it (Edelman et al., 1983).

The Quorn™ brand (http://www.quorn.com/) was launched in 1985 by Marlow Foods (UK). Quorn™ products contain mycoprotein from the filamentous fungus *F. venenatum*. The fungal biomass provides a texture that resembles meat products. Quorn™ may be the only SCP product exclusively used for human nutrition and has been extensively branded, marketed and sold for that purpose. The company was recently (2015) acquired by the Philippine instant noodles maker Monde Nissin Corp for 550 million pounds (http://www.reuters.com/article/quorn-ma-idUSL5N1204C720151001).

Spent brewer's yeast (*Saccharomyces cerevisiae*) have been sold for more than a century in yeast extracts such as Marmite® (Unilever and Sanitarium Health Food), Vegemite® (Bega Cheese Ltd.), Cenovis® (Gustav Gerig AG), and Vitam-R® (VITAM Hefe-Produkt GmbH). Yeast extracts provide a good source of five important group-B vitamins, but also protein. Another commercially available yeast, Torula (*Candida utilis*, renamed as *Pichia jadinii*), a widely used flavoring agent, is also high in protein. *Torula* was used in Provesteen® T, produced by the Provesta Corporation in the 1980s, along with similar products using *Pichia* and *Kluyveromyces* yeast (Hitzman, 1986). Torula is rich in the amino acid glutamic acid and for this reason it has been used to replace the flavor enhancer monosodium glutamate (MSG).

A process called “Pekilo” was developed in Finland to produce SCP for animal feed from the sugars present in sulphite liquor of paper mill effluents (reviewed in Ugalde and Castrillo, 2002). The filamentous fungus *Paecilomyces varioti* was grown on sugars, including pentoses, in the sulphite waste liquor or wood hydrolysates. There were two factories operating in Finland in Mänttä and in Jämsänkoski during 1982–1991, but as the cellulose mills ceased operations, these factories were also closed. Although, the product was sold as animal feed, it was also investigated as a supplement in meat products such as sausages and meat balls (Koivurinta et al., 1979). The Pekilo process strain is available from the VTT Ltd. culture collection (www.culturecollection.vtt.fi/).

Quorn™ and yeast spreads like Marmite® are produced from starch-derived glucose, while the Pekilo process used lignocellulosic sugars. In addition to these carbon sources, alkanes and methanol have been used for SCP production by yeast and filamentous fungi. Methylotrophic yeasts, for example *Komagataella pastoris* (previously *Pichia pastoris*), produce biomass and protein from methanol (Rashad et al., 1990). Industrial scale production has been carried out, e.g., by Phillips Petroleum Company. Their yeast produced 130 g (DW)/l biomass, with a productivity of more than 10 g l^−1^ h^−1^ (Johnson, 2013).

British Petroleum pioneered production of *Yarrowia lipolytica* SCP for animal feed from waxy n-paraffins from an oil refinery in the 1970's, building a pilot plant with up to 100 kton annual production capacity (Groenewald et al., 2014). Although the product itself was considered safe, the plant failed to get the required production permits because of environmental concerns (Bamberg, 2000). Combined with the high price of substrate resulting from the 1973 oil crises, this led British Petroleum to abandon its interest in SCP (Groenewald et al., 2014). *Yarrowia* SCP is now available on a smaller scale as Yarrowia Technology products (Yarrowia Equinox and Yarrowia GoodStart products) from Skotan S.A. in Poland (http://www.yarrowiatechnology.com/?lang=3). Although, oils and carotenoids are the most common *Yarrowia* products for human use (Groenewald et al., 2014), the American-based Nucelis also offers a protein rich Yarrowia Flour (https://www.nucelis.com/products.php?product=oils#circles).

Research and development on SCP with various fungal species is active and ongoing and may lead to novel products or production processes. For example, Zhao et al. (2013) described a process in which antibacterial peptides would be produced and secreted by *Y. lipolytica*, generating a high-value product, while the spent yeast could be used as SCP, since its protein content was high. Much of the current research focuses on the use of waste substrates such as sugarcane bagasse (e.g., *Penicillium janthinellum* with 46% protein, Rao et al., 2010), brewery's spent grains, hemicellulosic hydrolysate (e.g., *Debaryomyces hansenii*, White et al., 2008; *Kluyveromyces marxianus*, Aggelopoulos et al., 2014), whey (mixed yeast cultures, Yadav et al., 2014, 2016; *K. marxianus*, Aggelopoulos et al., 2014), and mixtures of other common food industry wastes such as orange and potato residues, molasses, and malt spent rootlets (*K. marxianus*, Aggelopoulos et al., 2014). Aggelopoulos et al. (2014) used solid state fermentation (SSF) rather than submerged cultivation and also noted that higher value products could be extracted prior to use of the protein-enriched residues as animal feed.

### SCP from bacteria

Bacteria also have a long history of use as SCP, particularly in animal feed. Some of the more commonly studied species have been reviewed by Anupama and Ravindra (2000), Rudravaram et al. (2009), and Nasseri et al. (2011) and Table 3 provides a list of more recent research on bacterial SCP.

**Table 3:** Recent reports of bacterial protein content on specific substrates for species investigated as potential sources of SCP.

**Organism**	**Substrate**	**Protein content (%)**	**References**
*Bacillus cereus*	Ram horn	68	Kurbanoglu and Algur, 2002
*Bacillus licheniformis*	Potato starch processing waste	38	Liu et al., 2014
*Bacillus pumilis*	Potato starch processing waste	46	Liu et al., 2013
*Bacillus subtilis*	Ram horn	71	Kurbanoglu and Algur, 2002
	Soy bean hull	12	Wongputtisin et al., 2012, 2014
*Corynobacterium ammoniagenes*	Glucose + fructose	61	Wang et al., 2013
*Corynebacterium glutamicum*^a^	n.a	57–70	Zhang et al., 2013
*Cupriavidus necator*	Synthetic growth medium	40–46	Kunasundari et al., 2013
*Escherichia coli*	Ram horn	66	Kurbanoglu and Algur, 2002
*Haloarcula* sp. IRU1	Petrochemical waste water	76	Taran and Asadi, 2014
*Methylococcus capsulatus, Ralstonia* sp.*, Brevibacillus agri, Aneurunibacillus* sp.	Methane (Natural gas)	67–73	reviewed in Øverland et al., 2010
*Methylomonas* sp.	Methane salt broth	69	Yazdian et al., 2005
*Rhizospheric diazotrophs* (whole microbial community)	Brewery wastewater	>55	Lee et al., 2015
*Rhodopseudomonas palustris*	Latex rubber sheet wastewater	55–65	Kornochalert et al., 2014

*n.a, not available*.

a *Commercial products, Prosin, and Protide, produced by CJ (Liao cheng) Biotech Co., Ltd., China*.

Bacterial SCP generally contains 50–80% protein on a dry weight basis (Anupama and Ravindra, 2000) and the essential amino acid content is expected to be comparable to or higher than the FAO recommendations (Erdman et al., 1977). Methionine content up to 3.0% has been reported (Schulz and Oslage, 1976), which is higher than that generally obtained in algal or fungal SCP. Similar amino acid composition is observed with methanol or methane grown bacteria (Øverland et al., 2010). As with fungi, bacterial SCP has high nucleic acid content (8–12%), especially RNA, and thus requires processing prior to usage as food/feed (Kihlberg, 1972; Nasseri et al., 2011; Strong et al., 2015). In addition to protein and nucleic acid, bacterial SCP provides some lipid and vitamins from the B group.

Imperial Chemical Industries developed a SCP (Pruteen) for animal feed from methanol, using the bacterium *Methylophilus methylotrophus*. Pruteen contained up to 70% protein and was used in pig feed (Johnson, 2013). Pruteen, however, could not compete with cheaper animal feeds that were available at the end of the 1970s and production was discontinued. Pruteen was produced from methanol, but methane is now gaining interest as a substrate for SCP. UniBio A/S (utilizing knowledge gained by Dansk BioProtein A/S) and Calysta Inc. have both developed fermentation technology to convert natural gas to animal feed protein by using methanotrophic bacteria. UniBio A/S uses a U-loop fermenter, to achieve a productivity of 4 kg m^−3^ h^−1^, producing UniProtein® with ~70% protein, which has been approved for use in animal feed (http://www.unibio.dk/company/subpage-1/). The U-loop fermenter is designed to enhance mass transfer rates of methane from the gas to the liquid phase, making more methane available for the bacteria (Petersen et al., 2017). Calysta Inc. opened a production facility for their product, FeedKind®, in the UK in 2016 and is partnering with Cargill to build a larger production facility in the U.S.A (http://calysta.com/commercialization/). FeedKind®, like UniProtein®, is used in animal feed. Methane is an interesting substrate, since it is a major by-product of cattle and pig farming (Philippe and Nicks, 2015), as well as being available from biogas production (landfills, waste). Excess methane is currently flared. VTT Ltd. is investigating the reactor design and options for coupling farm methane generation with the production of microbial oil and feed protein (http://www.vttresearch.com/media/news/protein-feed-and-bioplastic-from-farm-biogas) from the methanotrophic bacteria *Methylococcus capsulatus* (group I), *Methylosinus trichosporium* (group II), and *Methylocystis parvus* (group II).

As with SCP from fungi, other developments in the production of bacterial SCP focus on upgrading various waste substrates or valorisation of waste water treatment. Examples include the treatment of potato starch processing waste in a two-step process using *Aspergillus niger* to degrade fibres in the potato residue and *Bacillus licheniformis* to produce protein (Liu et al., 2014). Economic analyses indicated that the process could address not only the pollution problem of the starch industry, but also the shortage of protein for animal feed in China (Liu et al., 2014). Another example of simultaneous waste water management and SCP production was reported by Kornochalert et al. (2014) for rubber sheet factory waste. They demonstrated that the chemical oxygen demand, suspended solids and total sulfides in the waste water was reduced by the purple nonsulfur bacterium, *Rhodopseudomonas palustris*, to levels that met the guidelines for use as irrigation water in Thailand and that the biomass produced was suitable for SCP (Kornochalert et al., 2014).

Soy-bean hull has been fermented with *B. subtilis* to improve its nutritional value as a feed for monogastric animals (Wongputtisin et al., 2014).

Kunasundari et al. (2013) describe a novel secondary product, co-produced with bacterial SCP. They cultivated *Cupriavidus necator* in a large scale to produce biomass high in both protein and polyhydroxyalkanoate (PHA). This biomass was fed to rats. The feed was not only well-tolerated and safe for rats, but the rats also produced faecal pellets containing PHA granules, which enabled the purification of substantial amounts of PHA without use of strong solvents (Kunasundari et al., 2013).

## Processing of SCP

Depending on the substrate material and intended food/feed application, various processing steps are required prior to formulation of the final SCP product. In the following section we review the most relevant processing needs for SCP.

### Cell wall degradation in single cell protein products

Some SCP are used as whole cell preparations, while in others the cell wall may be broken down to make the protein more accessible. SCP, such as Quorn™, may be consumed without degradation of the cell wall, in which case chitin and glucan from fungal cell walls contribute fibre to the diet (Wiebe, 2004). SCP derived from *Euglena* does not require dirsuption since the cells have proteinaceous pellicles, rather than cell walls, making it more readily digestible.

Various methods have been used to disrupt the cell wall, including mechanical forces (crushing, crumbling, grinding, pressure homogenization, or ultra-sonication), hydrolytic enzymes (endogenous or exogenous), chemical disruption with detergents, or combinations of these methods (reviewed in Nasseri et al., 2011). Cell disruption may affect the quality and quantity of protein and other components in the SCP. Products such as Marmite® and Vegemite® are cell extracts, generated by heating the cells to 45–50°C long enough for intracellular enzymes to partially hydrolyse the cell wall; the proteins are also reduced to smaller peptides (Trevelyan, 1976; Ugalde and Castrillo, 2002).

### Nucleic acid removal in single cell protein products

Although algae generally have low nucleic acid content, the rapidly proliferating bacterial and fungal species have high nucleic acid (RNA) content. RNA content and degradation are affected by growth conditions, growth rate, and the carbon-nitrogen ratio (Trevelyan, 1976). When SCP is produced for human consumption, high nucleic acid content is a problem because ingestion of purine compounds derived from RNA breakdown increases uric acid concentrations in plasma, which can cause gout and kidney stones (Edelman et al., 1983). SCP with high nucleic acid content which is intended as animal feed is recommended only for feeding animals with short life spans (Strong et al., 2015). Gao and Xu (2015) and Xu (2015) have recently described methods for measuring the nucleotide content of complex SCP products.

Various methods to decrease the RNA content in SCP have been developed (Sinskey and Tannenbaum, 1975) and continue to be in use. Endogenous RNA degrading enzymes (ribonucleases) can be exploited in degradation of RNA, after activation with heat treatment (60–70°C) as used in the production of Quorn™ (Anderson and Solomons, 1984). Ribonucleases can also be added to the process or used as immobilized enzymes (Martinez et al., 1990; Hameş and Demir, 2015). Degraded RNA components diffuse out of the cells, but biomass loss (35–38%) also occurs. The process was improved by using higher temperatures (72–74°C) for 30–45 min, with less loss of biomass (30–33% loss; Ward, 1998). The temperature increase requires steam input, which is a cost factor, but heat is also needed for final treatment of the biomass at 90°C after the RNAse activation (Knight et al., 2001).

Alkaline hydrolysis and chemical extraction methods have also been studied. Viikari and Linko (1977) used an alkali treatment to reduce RNA in *P. varioti* biomass, used in for Pekilo-process, to below 2%. Treatment at 65°C, pH 7.5–8.5, to activate endogenous ribonuclease, also reduced the RNA content to <2%, while the protein content stayed at 50%.

## Safety of SCPs

As for any food or feed product, SCP needs to be safe to produce and use. Regulations exist in most regions to ensure that food or feed are safe for consumption (Bagchi, 2006). Typically these distinguish not only between food (for humans) and feed (for animals), but also between food (providing nutrition and potentially taste and aroma) and food additives (preservatives, colourants, texture modifiers, etc.), or feed and feed additives. Exact definitions may differ between regions, but international standards, regulated through the Joint FAO/WHO Expert Committee on Food Additives, apply to internationally traded products (WHO, 2017). Regulations differ depending on the intended purpose of the product, and although SCP is expected to be either food or feed (providing nutrition), some products may enter the market as additives (e.g., providing colour), rather than as SCP, even though protein is present in the product, limiting the extent to which they are added and their value as SCP. Coppens et al. (2006) summarised the European regulations related to food and food supplements, concluding that “the process of having ‘functional foods’ ready for the market is certainly a costly and time-consuming task,” but also that the process can be successful.

Smedley (2013) provides useful references to the specific regulations related to feed and feed additives in Brazil, Canada, China, the European Union, Japan, South Africa, and the United States, and the differences between the regulations in these regions. It should be noted that not all animals are regarded the same in all regions, thus pet food is regulated as feed in some areas, but not in others. Authorisation is required before sale of new feed or additives (Smedley, 2013).

Key concerns are the RNA content, toxins produced by microbes (production hosts or contaminants), potential allergy symptoms, and harmful substances derived from the feedstock such as heavy metals. Methods have been developed and are in industrial use to decrease the RNA content to acceptable levels, as discussed above.

The challenge of toxins is overcome by carefully selecting the production organism, the process conditions, and the product formulation. Some fungi produce mycotoxins and this makes them undesirable sources of SCP (Anupama and Ravindra, 2000). The effects of fungal toxins range from allergic reactions to carcinogenesis and death. Both humans and animals are affected, so mycotoxins cannot be tolerated in SCP for either human or animal consumption. Quorn™ mycoprotein underwent extensive testing for the presence of mycotoxins or other toxic compounds before being approved for human consumption (Wiebe, 2004). The particular strain of *F. venenatum* does not produce mycotoxins under production conditions, but the process is still monitored to ensure none are present. The initial safety testing for Quorn™ mycoprotein involved 16 years, with many more years required to gain approval for sale outside the UK (Solomons, 1986). *Y. lipolytica* is another fungus whose safety has been extensively assessed, demonstrating that it would be safe to use in a variety of food applications, including as SCP (Groenewald et al., 2014).

Bacteria may also produce toxins which limit their use as SCP. Toxins may be extracellular (exotoxins) or cell bound (endotoxins). For example, both *Pseudomonas* spp. and *Methylomonas methanica* produce high levels of protein and have been assessed for use as SCP. Both also produce endotoxins that cause febrile reactions (Rudravaram et al., 2009). These can be destroyed by heating. Further, a study on immunogenicity of SCP from *M. capsulatus* showed that the cell-free preparation (i.e., the cell wall is removed) did not cause immune responses in mice, although whole cell preparations did (Steinmann et al., 1990).

The use of varying waste types of raw materials for SCP production is appealing from the cost and sustainability point of view, but may be challenging from the safety perspective and the origin of the feedstock must be carefully considered. For example, Quorn™ is produced in a chemically defined medium from glucose (hydrolysed starch) in a well-defined process which meets GLP standards (Wiebe, 2002, 2004). Any product for human consumption which would be produced from biomass hydrolysates or waste streams would need to provide an equivalent safety record before finding approval in Europe or North America. In addition to the safety requirements associated with the use of waste-derived substrates for SCP, public perception and acceptance of waste-derived foods would be a key element to consider when implementing SCPs in human diets.

### Genetically modified organisms in SCP production—future possibilities

Use of genetically modified organisms (GMO) in food and feed has not yet found public acceptance in Europe, although there is more acceptance elsewhere in the world. As data regarding GMO consumption accumulates, they may gain further acceptance as protein sources become scarcer, particularly if a market develops for healthy or personalized nutrition. GMO yeast from bioethanol factories can already be used as cattle feed in some countries. Use of genetic elements from the host itself (self-cloning) often means that no foreign DNA is introduced.

Although, Goldberg (1988) discussed the prospect of using genetically engineered microbes as SCP in the 1980s as a means of improving process economics by producing co-products (e.g., an enzyme, organic acid, or antibiotic), the concept was not pursued and has only gained more interest and acceptance in recent years. A wide range of advantages in SCP products from genetic modification has been considered. For example, DuPont has genetically engineered a yeast to produce long-chain omega-3 fatty acids, which are essential to human health (Xie et al., 2015). Genome sequencing and genetic engineering also allow disruption of genes involved in toxin production and thus improved safety of some SCP products. Disruption of genes can be achieved by traditional mutagenesis and screening, but the process may introduce undesired mutations into the product, whereas genetic modification is quicker and more specific. This will be aided by new technologies, such as Clustered Regularly Interspaced Short Palindromic Repeats (CRISPR) that allows specific editing of the genome without introduction of new DNA. Strains which have been modified using CRISPR are not necessarily considered GMOs. CRISPR methodology can also eliminate the introduction of antibiotic resistance genes to the organism, avoiding concern about the spread of antibiotic resistance genes through the use of GMOs.

Carbon source metabolism is another target for improving SCP production processes, since the carbon source can be a major cost in SCP manufacture (Ugalde and Castrillo, 2002). Genetic engineering could broaden the range of substrates used by the production organism or increase the efficiency of their use, enabling the use of multiple feed stocks and ensuring that all potential carbon in the raw material is used. For example, Ren et al. (2016) introduced xylose fermentation capability from *Candida intermedia* to *S. cerevisiae* by genome suffling to enable ethanol production from glucose and SCP production from xylose, while Cui et al. (2011) introduced inulase to *Y. lipolytica*. Similarly, expression of one or more hydrolytic enzymes has improved use of polymeric substrates (Song et al., 2017). Cellulose, starch, or whey could be used in consolidated bioprocesses by an organism modified to produce the tailor-made enzyme cocktail suitable for the particular raw material. Organisms could also be engineered to have improved tolerance to acid, alkali or other compounds associated with specific substrates.

Genetic modifications could also increase the nutraceutical value of the biomass, either by optimising the amino acid composition or by increasing the content of specific vitamins (e.g., D-vitamin, B-vitamin, biotin), fatty acids, glutathione, etc. along with the protein. There is considerable scope for creating SCPs with tailor made, personalized, nutritional composition.

Genetic engineering may also provide new ways of harvesting the proteins for inclusion in food or feed. For example, modification to improve flocculation could reduce costs in collection of cells or the cells could be modified to have a set of cell wall degrading enzymes that would be activated by specific extracellular stimuli to provide proteins without cell walls. Similarly, ribonucleases could be designed to be activated at a specific time in conditions in which proteases would not be activated. Morphological characteristics could also potentially be engineered to provide specific organoleptic properties.

## Economic aspects

Development of SCP processes has always been driven by a need for protein, and this continues to be an important driver in the development of both old and new processes. The valorisation of readily available substrate and waste streams has also been a strong driver and continues to be so. SCP is frequently seen as a potential co-product that could strengthen the economic potential of an otherwise unprofitable biorefinery process, as well as a means of reducing the downstream processing costs required to dispose of process waste. Selling residual biomass as feed is preferable to selling as fertiliser. This is seen in the numerous publications and patents (not addressed in this review) in which specific waste products are converted to SCP and are assessed as food for specific animals. However, environmental concerns also now play a strong role in driving the development of novel SCP products. This is seen particularly in the processes which utilise greenhouse gases: algal SCP from CO_2_ and bacterial SCP from methane. Such processes are unlikely to be economically viable in the short term, since there are still many problems to overcome in large scale cultivation, but may survive where they are able to benefit from a green premium. In addition, environmental concerns, as well as economic concerns, are helping to drive the development of products from waste streams.

Apart from the environmental benefits, the key elements in estimating the economic viability of a SCP production process are total product cost, capital investment and profitability. Ugalde and Castrillo (2002) estimated that in fungal SCP production 62% of the total product cost would come from the raw material and 19% from the production process. According to Aggelopoulos et al. (2014), raw material costs vary from 35 to 55% of the manufacturing costs, whereas the operation costs, including labour, energy, and consumables take 45–55%. Utilising side-streams and waste biomass is sometimes viewed as a means to reduce the substrate costs, in cases when the substrate does not compromise the usability of the final product.

Scale is also important to the economic viability of SCP production. An empirical relationship exists between cost and scale of production. Continuous operations have been proven to be the most profitable ones and the majority of the SCP processes which have been implemented at industrial scale have been adjusted to continuous design (Ugalde and Castrillo, 2002). On the other hand, small scale, household production of some products may become feasible, in much the way that home yoghurt production or mushroom production has, and as has been suggested for plant cell nutrition without plants (Poutanen et al., 2017).

## Update on industrial production of SCP—players and capacities

Table 4, lists companies reported to produce or to have an interest in SCP, with website and patent information provided when available. A short description of some active companies is given below.

**Algaeon Inc**. produces β-glucan and whole cell products from the photosynthetic protist *Euglena gracillis*. Algaeon was started in 2011 and is based in the U.S.A.
**BlueBioTech Int. GmbH**, a microalgal biotechnology company, which has operated for more than 10 years, producing large quantities of *Spirulina* and *Chlorella*.
**Calysta Inc**. was founded as a private company in 2011. It produces FeedKind® from methane at a pilot facility in the UK, and began distributing commercial samples in 2017. It plans to open a larger facility (producing up to 20,000 tonnes per year) in the U.S.A. in 2019.
**Cangzhou Tianyu Feed Additive Co., Ltd** is a manufacturer and trading company located in Hebei, China since 2004. Their main products are Yeast Powder, Choline Chloride, Betaine, and Allicin having markets in Southeast Asia, Eastern Asia, Oceania, South Asia, and South America. The company employs 50 people and their total revenue is 5–10 million US$.
**CBH Qingdao Co., Ltd** has been an established company for decades supplying a range of ingredients and additives for feed and food industries. They can supply products which meet FAMI-QS, ISO, GMP, KOSHER, and HALAL standards.
**Cyanotech Corporation** is one of the world leading producers of *Spirulina* with sales in the US and 30 other countries. Their turnover in 2016 was almost 32 million US$. FDA has given GRAS status for Cyanotech's *Spirulina* as a food ingredient.
The progenitor of **Earthrise**, Proteus Corporation was founded in 1976. They produce *Spirulina* with GRAS status. They are GMP certified and have Food Safety System Certification (FSSC) 22000:2011.
**E.I.D Parry Ltd., Parry Nutraceuticals Division** is part of the 4.4 billion US$ Murugappa Group. They use micro-algal technology to produce nutraceuticals like *Spirulina* and *Chlorella*. Their products are sold in more than 40 countries and their main markets are in North America, Europe, South East Asia, and the Far East.
**Euglena Co. Ltd**. was founded in Japan in 2005. Amongst other products derived from *Euglena gracillis*, Euglena Co. Ltd. is developing de-fatted Euglena as a source of protein-rich animal feed.
**KnipBio** was founded in 2013 in the U.S.A. with a focus of providing affordable feed for aquaculture. They produce KnipBio Meal from methanol using a methylotrophic bacterium and plan to start commercial production in 2018.
**Lallemand Inc**. is a Canadian company specializing in the development, production, and marketing of yeast and bacteria. There are two major groups in the company: the Yeast Group (based in Montreal, Canada) and the Specialties Group (based in Toulouse, France). They produce SCP for human consumption (LBI, Lake States®, Engevita™) from the yeast *S. cerevisiae* and *Torula*.
**LeSaffre** produces yeast (*S. cerevisiae*) and yeast derived products including SCPs such as Lynside® Nutri, Lynside® ProteYn and related products (Lesaffre Human Care products), as well as yeast-based flavour ingredients (Biospringer products). The company has 7,700 employees and more than 80 subsidiaries in over 40 countries. Their products and services are sold in more than 180 countries and their turnover was ~1.6 billion € in 2013.
**Marlow Foods Ltd** produces the mycoprotein Quorn™. The Quorn development project started already in the 1960s, when they started to look for a microbial protein source that humans would find enjoyable. Quorn is classified as a safe, well-tolerated food by regulatory bodies across the world, including FDA, and the UK's Food Standards Agency (FSA). The company was acquired by Monde Nissin Corporation in the Philippines for 831 million US$ in 2015.
**Nucelis Inc**. was founded in 2010 in the U.S.A., but became a subsidiary of **Cibus Global** in 2014. Along with squalene, vitamin D and nutritional oils, Nucelis Inc. is developing high protein flour from the yeast *Yarrowia*.
**Nutrinsic** is based in the USA, with subsidiaries in China. Nutrinsic focuses on the use of waste waters from the food, beverage and biofuel industries to generate feed and fertiliser products. They market a SCP for animal feed called ProFloc™, which is described as having a protein content around 60%. They opened their first USA production facility in 2015, using waste water from a local brewery.
**Tangshan Top Bio-Technology Co., Ltd** is a manufacturer and trading company located in Hebei, China (Mainland). Their main products are: brewer's yeast, autolyzed yeast, yeast cell wall and yeast extract, including a 100% natural, non-GMO, pure yeast powder as animal feed additive for 1,100–1,250 US$ per ton and a production capacity of 15,000 tons per year per production line. The company was established in 2009 and has ~200 employees. Their main markets are in China, Eastern Asia, Western Europe, Southeast Asia and Mid East, with 40–50% of their products exported.
**TerraVia Holdings, Inc**. is a publicly held American company which focuses on providing ingredients for food and care products from eukaryotic algae. TerraVia appeared in 2016, but is derived from Solazyme Inc. which was founded in 2003. TerraVia uses traditional stirred tank reactors to cultivate its algae.
**UniBio A/S, Denmark** is an SME that owns rights to a unique fermentation technology—the U-Loop technology, which enables natural gas to be converted into a high protein product—UniProtein®. UniProtein® has a protein content of ~71% and can be used in feed for animals. UniBio A/S was established in 2001.
**Unilever** produces yeast extract Marmite® from brewer's spent grain. The number of employees is around 169,000 and the turnover of the company was $52.7 billion in 2016.
**Vega Pharma Ltd** is located in Zhejiang, China—the Vega Group is developing, manufacturing, and marketing pharmaceuticals, nutritional ingredients, animal health products, and probiotics. They offer a SCP, with up to 65% protein and containing relatively high threonine levels, for animal feed as a by-product of monosodium glutamate production.

**Table 4:** Industrial establishments involved in SCP production.

**Company, Country**	**Microorganism**	**Substrate**	**Patent, web site/references**
Algaeon^b^	*Euglena gracilis*	CO_2_	http://algaeon-inc.com/
Amoco (BP), USA^a^	*Candida utilis*	Ethanol	Rudravaram et al., 2009
Bega Cheese Ltd ^b^	*Saccharomyces*	Wheat	http://www.smh.com.au/business/bega-snaps-up-vegemite-as-part-of-460m-deal-20170118-gtu7wk.html
Bellyyeast, FR^c^	*Kluveromyces*	Whey	Rudravaram et al., 2009
BlueBio Tech Int. GmbH, DE^b^	*Spirulina, Chlorella* sp.	CO_2_	www.bluebiotech.de
Blue Green Foods^b^	*Aphanizomenon flos-aquae*	CO_2_	https://bluegreenfoods.com/
Calysta Inc. UK^b^	Soil microbes	Methane	www.calysta.com
Cangzhou Tianyu Feed Additive Co. Ltd., CN^b^	Yeast powder (n.a.)	n.a.	www.cztymy.com
CBH Qingdao Co., Ltd, CN^b^	Bacterial fermentation (n.a.)	n.a.	www.cbhcn.com
Cyanotech, USA^b^	*Spirulina platensis*	Sodium bicarbonate, CO_2_	www.cyanotech.com/ US19920959649, US19960641159
E3Live^b^	*Aphanizomenon flos-aquae*	CO_2_	https://www.e3live.com/
Earthrise, USA^b^	*Spirulina* sp.	CO_2_	www.earthrise.com/
E.I.D Parry Ltd., Parry Nutraceuticals Division, IN^b^	*Arthrospira platensis, Chlorella vulgaris*	CO_2_	www.parrynutraceuticals.com/#
Euglena Co. Ltd.^b^	*Euglena*	CO_2_	http://www.euglena.jp/en/
FEBICO^b^	Spirulina, *Chlorella*	CO_2_	http://www.febico.com/
Flint Hills Resources^b^	*Saccharomyces cerevisiae*	Corn	https://www.fhr.com/newsroom
Hainan Simai Enterprising Ltd.^c^	Spirulina	CO_2_	
Imperial Chemical Industries, UK (now: AkzoNobel, NL)^a^	*Methylotrophus*	Methanol	Rudravaram et al., 2009; Johnson, 2013
IFP, FR^c^	*Candida tropicalis*	n-alkanes	Rudravaram et al., 2009
Kanegafuichi, JP^c^	n.a.	n.a.	Rudravaram et al., 2009
Klamath Valley Botanicals LLC^b^	*Aphanizomenon flos-aquae*	CO_2_	http://klamathvalley.com/
KnipBio^b^	*Methylobacterium extorquens*	methanol	www.knipbio.com
Lallemand Inc., CA^b^	Yeast and bacteria	n.a.	www.bio-lallemand.com
LeSaffre, FR^b^	Yeast	n.a.	http://www.lesaffre.com/
Liquichemica, IT^b^	*Candida maltosa*	n-alkanes	Rudravaram et al., 2009
Marlow Foods Ltd. UK^b^	*Fusarium venenatum*	Glucose syrup	www.quorn.co.uk
Mayanmar Spriulina Factory^c^	Spirulina	CO_2_	
Mondelez Int.^b^	Yeast	brewer's spent grain	http://www.mondelezinternational.com/
Nucelis^b^	*Yarrowia lypolitica*	n.a.	https://www.nucelis.com/
Nutrinsic^b^	*Bacteria*	starch, brewing, other waste waters	http://nutrinsic.com/ ZA201003590
Phillips Petroleum Company USA (Ohly)^b^	*Pichia* sp. *Torula* sp.	Sugar feed stock	Rudravaram et al., 2009
Qingdao Zhongtai Poultry Ind. Professional Cooperatives, CN^c^	Lactic acid bacteria	Whey, Whey & soybean meal, wheat & rice bran, beer lees, jujube, urea	CN102894183 CN102987056
Roquette Klötze GmbH & Co^b^	*Chlorella*	CO_2_	http://www.algomed.de/en/homepage/
Shanghai Gentech Ind. Group Co. Ltd. CN^d^	n.a.	n.a.	CN103843971
Shanghai Tramy Green Food Co, CN^b^	*Aspergillus oryzae, Saccharomyces cerevisiae, Tricoderma* sp.	Bean dregs and soybean processing water: Soybean dregs, Bean waste water	CN103098979 CN103156051
Skotan S.A., PL^b^	*Yarrowia lipolytica*	n.a.	http://www.skotansa.pl/
Skystone Feed Yixing Co, CN^b^	*Aspergillus niger*	Blue-green algae	CN103749957
Tangshan Top Bio-Technology Co., Ltd., CN^b^	*Saccharomyces* sp.	n.a.	www.tuopobio.com
Taiwan Chlorella Manufacturing Co.^c^	*Chlorella*	CO_2_	
TerraVia, USA^b^	Alga	n.a.	http://algavia.com/
UniBio A/S, DK^b^	Methanotrophic bacteria	Natural gas	www.unibio.dk PA199900690
Unilever^b^	Yeast	brewer's spent grain	www.unilever.com
Vega Pharma Ltd., CN^b^	Bacterial	n.a.	www.vegapharma.com

*n.a. not available*.

a *Company inactive or has merged or been taken over by another company, with a new name*.

b *Active in SCP production*.

c *Current activities in SCP unknown*.

d *Active through partners*.

## Recent patents (2001–2016)

Recent patents (2001–2016) related to SCP production with algae, fungi, bacteria and mixed microbial populations are listed in Tables 5–8. Some of the patents owned by industrial operators are also shown in Table 4. The number of patents related to the use of algae, bacteria, yeast, or mixed populations is relatively evenly divided. Many patents have also been filed in which microbial biomass forms a component of a feed mixture which is intended to provide protein and other nutrients to fish or farmed animals. These have not been included in Tables 5–8, since it is not clear how much protein is provided by the microbe and how much by other components such as soy, bean, or fish meal.

**Table 5:** Patents related to the production of SCP from algae during 2001–2016^*^.

**Patent number**	**Title**	**Publication date**	**Assignees and Inventors**
WO2017050917 (A1)	Novel method for the culture of unicellular red algae	2017-03-30	**Fermentalg**; Cagnac, O., Richard, L., Labro, J.
CN105861379 (A)	Method for preparing algae powder by drying peculiar smell-free blue algae	2016-08-17	**Jiangnan U**.; Yang H., Ping S., Wang T., Wang W., Zhang L.
WO2016092583 (A2)	Method for growing microalgae, and device for implementing said method	2016-06-16	**Lavanga V**.; Lavanga V. and Farne S.
CN105483014 (A)	Production technology for high-density culture of Chlorella by utilizing fermentation method	2016-04-13	**Qingdao Kehai Biological Co., Ltd**.; Li Y., Xia X., Xu J., Cui J., Li X., Wang D., Chi S., Sun H., Zhao S., Zhao S., Wang B., Cui X.
US2016021923 (A1)	High-Protein Gelled Food Products Made Using High-Protein Microalgae	2016-01-28	**Solazyme Inc**.; Paulsen S., Klamczynska B., Plasse K., Bowman C.
AU2015271929 (A1)	Novel microalgal food compositions	2016-01-21	**Solazyme Inc**.; Brooks, G., Franklin, S., Avila, J., Decker, S. M., Baliu, E., Rakitsky, W., Piechocki, J., Zdanis, D., Norris, L. M.
US2015374012 (A1)	High-Protein Food Products Made Using High-Protein Microalgae	2015-12-31	**Solazyme Inc**.; Klamczynska B., Echaniz A., Zhu R.
US2015320086 (A1)	Microalgae Meal	2015-11-12	**Solazyme Inc**.; Piechocki J., Hansen S. L., Loy D. D., Genther-Schroeder O., Stokes R., Van Emon M.
CN104946536 (A)	Isochrysis zhangjiangensis culture method	2015-09-30	**Dalian U**.; Yang H., Wang X., Xu P., Pan Y., Yu Y.
WO 2013086302 (A1)	Fractionation of proteins and lipids from microalgae	2013-06-13	**Old Dominion U. Research Foundation**; Kumar, S., Hatcher, P. G.
US 20100303990 (A1)	High Protein and High Fiber Algal Food Materials	2010-12-02	**Solazyme, Inc**.; Brooks, G., Franklin, S., Avila, J., Decker, S.M., Baliu, E., Rakitsky, W., Piechocki, J., Zdanis, D., Norris, L.M.
TW20070136127	SCP producing method	2009-04-01	**Zen U Biotechnology Co Ltd**; Chen C.H., Lu C.Y.
KR20040069402	Production of rice cake and noodles, which contain well balanced nutrients and have improved product quality, using fresh-water chlorella	2004-09-21	**Kim Soon Bok**; Kim S. B.
WO 2001005414 (A1)	Algae protein polysaccharide extraction and use thereof	2001-01-25	**Qing Qi, Jian Ding**; Qi, Q., Ding, J.

* The search was carried out in Espacenet and focused on patents from companies with known activities with SCP from algae, in addition to use of keywords (alga^*^ AND “single cell protein”).

**Table 6:** Patents related to the production of SCP from yeast or filamentous fungi during 2001–2016^*^.

**Patent number**	**Title**	**Publication date**	**Assignees; Inventors**
CN106011198A	A prepared from lignocellulosic biomass in agroforestry synthetic starch, cellulosic ethanol and the method of single cell protein	2016-10-12	**Tonglu Tang Won Biotechnology Co. Ltd**.; Ma, H., Ma, Y., Xu, X., You, C., Zhang, W., Zhang, Y.
CN105884104A	A membrane distillation—nanofiltration / reverse osmosis process as a combination of a high concentration of useful components of fermentation waste recycling and waste water purification method	2016-08-24	**Beijing Forestry U**.; Zhang, L., Qu, D., Kang, Y., Feng, L., Cheng, X.
CN105861343A	A preparation of high-lysine with methanol Single Cell Protein H. polymorpha yeast and its application	2016-08-17	**Nanjing U. of Technology**; Dai, Z., Dong, W., Jiang, M., Ma, J., Wu, R., Zhang, W.
CN105505803A	Saccharomycete and application thereof	2016-04-20	**Huayan Medical Res. Inst. Co. Ltd**.; Wang X.
CN104054907A	Preparation method for protein feed	2014-09-24	**Hunan Agricultural Products Proc. Inst**.; Fu, F., He, J., Li, G., Liu, W., Shan, Y., Su, D.
CN103881932A	Method for producing single-cell protein from water-chestnuts by virtue of fermentation	2014-06-25	**U. of Hezhou**; Pan, B., Su, L.
CN103849575A	Production method of single-cell protein	2014-06-11	**Nanchang University**; Gao, Z., Huang, H., Liu, Y., Ma, X., Ruan, R., Wan, Y., Wang, Y., Wu, X., Zheng, H.,
CN103844269A	Method for producing rice and noodle food through single-cell protein nutrient solution prepared by fruit and vegetable fermentation	2014-06-11	**Lan Jingmo**; Lan J.
CN103749957A	Preparation method for blue-green algae single-cell protein feed	2014-04-30	**Skystone Feed Yixing Co. Ltd**.; Liu, F., Xue, H., Yu, J., Zou, S., Zou, Y.
CN103627645A	Method for preparing carotenoid-enriched yeast single-cell protein by using bean curd yellow water for fermentation	2014-03-12	**Shenyang Chemical Technology U**.; Chu, C., Li, H., Liu, G., Yang, H.
CN103361279A	Method for producing single-cell protein by using pulping waste liquor	2013-10-23	**Beijing Yingli Shengke New Material Technology Co. Ltd**.; Zhuang, Y., Yin, Y., Huang, Y., Fan, H., Chen, T.
CN103275875A	*Trichoderma koningii*, and compound microbial agent composition and application thereof	2013-09-04	**An Jianping; Ma Weichao**; An, J., Ma, W.,
CN103271233A	Method for producing single-cell protein feed by means of fermentation of gallnut residues	2013-09-04	**Jishou U**.; Tian, H.
CN103103137A	*Saccharomycete* for hydrolyzing starch, producing lactic acid and fixing nitrogen, as well as application thereof	2013-05-15	**U. Xi An Jiaotong**; Huang, J., Lin, J., Liu, J., Long, J., Wang, L., Wu, Y., Yang, S.
CN103045494A	*Pichia pastoris* for efficiently converting methanol to produce single cell protein and application of *Pichia pastoris*	2013-04-17	**Coal Biochenmical High Tech Engineering Co. Ltd. Yima Coal Group**; Cao, M., Fan, C., Gou, W., Hu Yuansen; Qiao, G., Tian, Y., Wang, L., Wang, X., Wei, H., Xu, Z., Zhang, G., Zhang, Y., Zhu, G.
CN103027183A	Feed production method	2013-04-10	**Anhui Normal U**.; Liu, A., Lu, C., Peng, P., Zhang, Y.
WO13021138A2	Yeast flakes enriched with vitamin D2, compositions containing same, method for preparing same, uses thereof, and device for implementing the method	2013-02-14	**Lesaffre and Cie**; Fuentes, J.L., Knobloch, C., Mouly, I.
CN102911882A	*Rhodosporidium kratochvilovae* and application of same in preparation of carotenoid and single-cell protein	2013-02-06	**Nanjing Normal U**.; Chen, Y., Liu, J., Sun, H., Wei, M., Zhao, Y.
CN102815795A	Method for processing starch wastewater as well as product and application thereof	2012-12-12	**Guangxi U. for Nationalities; Nanning Suboante Biochemical and Scient. Co. Ltd**; Huang, J., Lan, L., Lan, P., Li, M., Liao, A., Shi, M., Wu, R., Xie, T.
US2010303778A	Composition for human and/or animal nutrition, uses thereof and yeasts	2010-12-02	**D'Auvergne Clermont 1 U., Du Droit et de la Sante Lille 2 U., Lesaffre and Cie**; Vandekerckove, P., Sivignon, A., Poulain, D., Desreumaux, P., Darfeuille, M.A., Simon, J.L., Pignede, G. Pierre uille Michaud Arlette
US2010092611A	Coated dried active yeasts and food products containing the same	2010-04-15	**Danstar Ferment Ag, Lallemand Sas**; Brouzes, J., Degre, R.
US2009232942A	Yeast preparations with improved antioxidant properties and uses thereof	2009-09-17	**Danstar Ferment Ag, Lallemand Sas**; Baulez, M., Degre, R., Forbes, W., Zhang, Z.
CN101386817A	Method for producing yeast single cell protein by blue algae fermentation	2009-03-18	**U. Jiangnan**; Yang, H., Li; K., Zhang, L., Wang, W.
US2004185162A	Edible fungi	2004-09-23	**Marlow Foods Ltd**.; Blanchard, R., Finnigan, T.J.A.

* *Search was made using the PatBase Express (www.patbase.com) database with the basic search, using key words “single cell protein” and checked manually*.

**Table 7:** Patents related to the production of SCP from bacteria during 2001–2016^*^.

**Patent number**	**Title**	**Publication date**	**Assignees & Inventors**
US201519779A	Microorganisms for the enhanced production of amino acids and related methods	2015-07-16	**Calysta Inc;** Doss, B.D., Giver. L.J., Luning. E.G., Regitsky. D.D., Saville. R.M., Resnick, S.M., Silverman. J.A.
CN104489281A	Method for preparing single-cell protein feed additive by processing wastes	2015-04-08	**Jiangsu Qianyaotang Traditional Chinese Medicine Res. Inst. Co. Ltd**.; Zhang K., Zhang Z.
CN104472867A	Method for preparing single-cell protein feedstuff by utilizing waste liquid in production of ginkgo leaf extracts and application	2015-04-01	**Jiangsu Qianyaotang State Medical Res. Inst. Co. Ltd**.; Zhang, K., Zhang, Z.
CN104450513A	Full-automatic factory full-wave band closed circulating water real-time monitoring breeding device	2015-03-25	**Zhu Zuyang**; Zhu Z.
US2015044327A	Methylotrophs for aquaculture and animal feed	2015-02-12	**Knipbio;** Marx, C. J., Feinberg, L.F.
US2016333384A	Carbohydrate-enriched recombinant microorganisms	2015-01-16	**Calysta Energy Llc; Calysta Inc;** Giver, L.J., Mueller, J., Regitsky, D.D., Saville, R.M., Silverman, J.A.
CN103918874A	Method for improving quality of manioc wastes by using mixed fermentation technology	2014-07-16	**U. of Sichuan Agricultural**; Chen, X., Jia, G., Liu, G., Tang, J., Tang, X., Zhao, H.
CN103484395A	Bacterial strain used for preparing single-cell protein from methanol, and applications of bacterial strain	2014-01-01	**Henan Coal Chemical Ind. Group Inst. Co**.; Chao, Y., Jiang, Y., Jiao, Z., Li, N., Li, W., Li, Z., Song, C., Su, M., Wang, Y., Wei, L., Zhang, X.
CN103156051A	Method using composite bacterium to ferment bean dregs to manufacture protein feed	2013-06-19	**Shanghai Tramy Green Food Co. Ltd**.; Li, L., Shen, J., Yuan, H.
US2014323694A	Multiphase porous flow reactors and methods of using same	2013-05-23	**Calysta Energy Inc;** Gosse, J.L., Harwood, T., Thust, S., von Keitz, M.G.
CN102987054A	Production process of fermented feed	2013-03-27	**Qingdao Tianrui Ecological Technology Co. Ltd**.; Qu T.
CN102987056A	Fermentation method for whey fermentation liquor for fermenting feed	2013-03-27	**Qingdao Zhongtai Poultry Ind. Professional Cooperatives**; Qu T.
CN102978271A	Method for producing carotinoid and single-cell protein via transforming cellulose pyrolytical liquid and levo-glucosan through photosynthetic bacteria	2013-03-20	**Nanjing Normal U**.; Chen, Y., Liu, J., Sun, H., Wei, M., Zhao, Y.
CN102972622A	Preparation method of whey fermented forage	2013-03-20	**Qingdao Tianrui Ecological Technology Co. Ltd**.; Qu T.
CN102960538A	Unicellular protein feed prepared from fermented dragon fruit peel and production method of unicellular protein feed	2013-03-13	**Guangxi Zhuang Autonomous Region Ct for analysis and test research**; Lu, A., Mo, J.
CN102894183A	Preparation method of whey fermented feed	2013-01-30	**Qingdao Zhongtai Poultry Ind Professional Cooperatives**; Qu T.
CN101507469A	Preparation method of composite zymoprotein	2009-08-19	**Jiangxi Purun Mechanical Co. Ltd**.; Yang, T.,
US2009114602A	Biosolids-based food additive for animal feed, methods of production, and business application thereof	2009-05-07	**Nutrinsic Corp; Oberon Fmr Inc; Procell Investments Ltd**; Logan, A.J., Swenson, R.P. Jr., Seth, S.T.
US2004241790A	Method of fermentation	2004-07-09	**Cockbain Julian; Norferm Da; Statoil Asa; Statoilhydro Asa; Bioprotein As; Calysta As;** Eriksen, H., Joergensen, L., Strand, K.
US2003138878A	Method	2003-07-24	**Golding L., Johannessen A., Kleppe G., Larsen J., Moen E., Norferm Da Stavanger, Statoil ASA**; Moen, E., Larsen, J., Kleppe, G., Johannessen, A.

* *Search was made using the PatBase Express (www.patbase.com) database with the basic search, using key words “single cell protein” and checked manually*.

**Table 8:** Patents related to the production of SCP from mixed microbial populations (bacteria and/or yeast and/or algae) or in which the microorganism was not specified during 2001–2016^*^.

**Patent number**	**Title**	**Publication date**	**Assignees and Inventors**
WO17083351	Heterologous expression of taurine in microorganisms	2017-05-18	**Knipbio Inc**; Feinberg, L.F., Marx, C.J., McAvoy, B.D., Wall, M.A., Smith, D.R., Pujol-Baxley, C.J.
CN106035985A	A mixed culture method for liquid fermentation of rice wine processing wastes to produce single cell protein	2016-10-26	**Tongji U**.; Chen, Y., He, Q., Pang, W., Xie, L., Zhou, Q.
WO16161549A	Method for producing aerobic single-cell protein by using autolysis process	2016-10-13	**Taizhou Icell Bio Tech Co. Ltd**.; Song, J., Xu, J.G., Zhang. X., Zhao, W.
CN105907801A	The use of continuous production of potato waste dietary fiber, alcohol and method of single cell protein	2016-08-31	**Shanxi Agricultural U**.; Hao, L., Zhang, H.
CN105176850A	Method for production of aerobic single-cell protein by enzymolysis tank autolysis process	2015-12-23	**Taizhou Aixier Biological Science and Technology Co. Ltd**.; Song, J., Xu, J.G., Zhang, X., Zhao, W.
CN105166322A	Method for high yield production of aerobic single-cell protein by autolysis process	2015-12-23	**Taizhou Aixier Biological Science and Technology Co. Ltd**.; Song, J., Xu, J.G., Zhang, X., Zhao, W.
CN104893976A	Method for producing aerobic type single-cell protein through autolysis process	2015-09-09	**Taizhou Aixier Biotechnology Co. Ltd**.; Song, J., Xu, J.G., Zhang, X., Zhao, W.
CN104489281A	Method for preparing single-cell protein feed additive by processing wastes	2015-04-08	**Jiangsu Qianyaotang Traditional Chinese Medicine Res. Inst. Co. Ltd**.; Zhang, K., Zhang, Z.
CN104381607A	Phycomycete complex fermented feed additive and preparation method thereof	2015-03-04	**U. of Yantai**; Lin, J., Sun, L., Zuo, Z.
CN104186431A	High-density Artemia breeding method with single-cell protein	2014-12-10	**Tiajin Ocean Pal Carol Biotech. Co. Ltd**.; Chai, C., Qian, H.
CN104171265A	Processing technology of single-cell protein from distilled spirit lees	2014-12-03	**U. of Sichuan Agricultural**; Jiao, H., Wang, L., Wang, Z., Xue, B.
CN103843971A	Method for preparing single-cell protein from biological slurry	2014-06-11	**Shanghai Gentech Ind. Group Co. Ltd**.; Hu, F., Song, J.
CN103695324A	Single-cell protein production method from waste watermelon peel	2014-04-02	**Hezhou U**.; Pan, B., Li, C.
CN103098979A	Method for producing single-cell protein feed by utilizing bean product waste water	May 15, 2013	**Shanghai Tramy Green Food Co. Ltd**.; Li Li; Shen Jianhua; Yuan Hui
CN102113622A	Straw and pot ale mixed fermented feed and production method thereof	2011-07-06	**Nankai U**.; Liu, Y., Wu, W., Tong, S., Wang, P., Ju, M., Liu, J.
CN101965900A	Method for producing probiotic single cell protein by using soybean molasses	2011-02-09	**Haflru; Heilongjiang Bayi Agricultural U**.; Li, D., Liu, Y., Gao, Y.
CN101962919A	Novel catalytic reactor method for manufacturer-grade paper pulp, native lignin and single cell protein	2011-02-02	**Jose Antonio Rodriguez Rivera**; Rivera J.A.R., O'Flynn, K.A.
CN101731450A	Preparation method of single cell protein feed by taking acetone butanol fermentation wastewater as raw materials	2010-06-16	**Jiangsu Lianhai Biolog Technology Co. Ltd**.; Tang, B., Wang, Z., Wen, Z.
WO2007065241A1	A novel catalytic reactor process for the production of commercial grade pulp, native lignin and unicellular protein	2007-06-14	**Kelly Anthony O'Flynn and Jose Antonio Rodriguez Rivera**; O'Flynn, K.A.Rivera J.A.R.,

* *Search was made using the PatBase Express (www.patbase.com) database with the basic search, using key words “single cell protein” and checked manually*.

Industries and universities in China have been particularly active in filing patents related to SCP in recent years, with about 70% of patents awarded since 2001 having been filed in China. In China, there has been a strong emphasis on the production of SCP by fermenting agricultural or food residues with bacteria, yeast and mixed populations. SCP production is thus often combined with bioremediation and waste processing.

Several important patents related to the use of C1 compounds such as methanol and methane were filed before 2001 and have not been included in this review. However, there were two new patents on the production of SCP from methanol and six on producing SCP from methane (Table 7). SCP from algae also continues to generate patents, with formulation of products attracting attention as well as continued developments in the cultivation methods (Table 5).

## Concluding remarks

As seen in Table 4, there are a wide range of industries involved in SCP production, some producing SCP as a by-product of other processes, and others which focus primarily on SCP. SCP from filamentous fungi and yeast continues to dominate the established markets, particularly when considering SCP for human consumption. Yeast SCPs have a long history of use, which facilitates their continued acceptance in the market. SCP for humans from filamentous fungi, however, is likely to remain restricted to *F. venenatum* (Quorn™) and solid-state fermentations with other food fungi, because of the risk of mycotoxins and the long path to regulatory acceptance. Yeast also have a long history of use as supplements to the feed industry. Much of the fungal SCP provided for animal feed is a byproduct of the food and beverage industries and of biorefineries, in which the fungus first acts as the biocatalyst to create the main product and then provides protein-enriched residues which are sold as feed. Fungal SCPs offer the advantages of familiarity, with well-established processing approaches, and availability. The main barrier is in the introduction of SCP from new species, which generate academic and patent interest, but which are difficult to bring into the market.

Algae also have well-established markets for both food and feed applications, although these are not traditionally focused on algae as SCP, but rather as food supplements providing omega-3 fatty acids, carotenoids and vitamins, with protein as a corollary benefit. Since products have been treated as supplements or colorants, the regulatory requirements are different than those for direct food or feed use, facilitating the introduction of new species for potential products. Algal products typically have flavours which may limit the amount a person would want to consume, reducing the need for extensive processing to reduce RNA, but also limiting the amount of protein provided to the diet. However, several of the young SMEs which have entered the market are developing processes to produce low-flavour products which could expand the contribution of algal protein in human diets. Algal SCP offers the advantages of providing healthy lipids along with the protein, while potentially consuming CO_2_. It has the benefit of being seen as environmentally friendly and very “green.” The main barriers are cost of production and the need for novel formulation to make it acceptable for humans. Algal SCP is likely to be strong in the feed industry, if the production costs can be reduced.

Bacterial SCP is primarily restricted to the feed industry, if not including cyanobacterial products with non-photosynthetic bacteria. Some bacterial SCP is currently a by-product of other industries such as monosodium glutamate production, and this type of feed product is expected to increase with the expansion of biorefineries, as with yeast. However, the most interesting current bacterial SCP developments relate to the use of methane as a carbon source. Although, the use of methane to produce bacterial biomass is not new, the drivers pushing developments have shifted from methane as a cheap carbon source to bacteria as a means of reducing green-house-gas emissions and the potential integration of feed production with animal farming. The low solubility of methane, coupled with low growth rates of the bacteria, poses a strong barrier to success in this area. However, young SMEs like UniBio and Calysta Inc. believe that the barriers can be overcome. Bacterial SCP, other than from methane, offers advantages in high production rates, but is disadvantaged by low familiarity and high nucleic acid content which adds to the processing costs.

SCP initially gained importance in human nutrition during times of war, when traditional sources of protein became scarce. It again became of interest during the latter half of the twentieth century because of concern about meeting the protein demands of the world's ever increasing population. These concerns were global, but when we consider current interest in SCP, we observe that the countries now driving research and development of new SCP are generally those with large populations (e.g., China and India) and problems with malnutrition. Most recent patents related to SCP have been filed from China, indicating the importance of SCP for future food and feed there. Fast growth of SCP products can be expected in China and perhaps in the whole of Asia. Development of algal SCP forms an exception to this observation—since many companies have been established around the world in recent years to develop products which can exploit the current excess availability of CO_2_. The drivers for development of algal SCP are thus somewhat different from those for the development of bacterial and fungal SCP. Production of SCP from methane shares this environmental concern and opportunity with the algal developments.

An increase in biorefinery processes, as part of the expansion of the bio-economy and circular economy concepts, also acts as a driver for the development of SCPs for use as animal feed, since conversion of waste material to animal feed offers better returns on investment than burning residual microbial biomass or utilising it as fertiliser. Regulatory clearance is still needed for use of novel products in animal feed, but this differs from that needed for human food use, and a wider range of substrates are considered acceptable when the product is intended for animal use. Thus, greater expansion in available SCPs for animal feed than for human food can be expected. None-the-less, there is a growing appreciation of the inefficiency of converting plant biomass into SCP which is fed to animals, rather than directly to humans, which will push development of safe SCP as food also.

In the west, interest in healthy diets and novelty food is helping to drive a new interest in SCP, while also blurring the edges of what products might be included in SCP. Cell cultures of both plant and animal cells may contribute to food supply in the future (Poutanen et al., 2017), but do not conform to the definition of SCP as being derived from microbial cells. In addition, the forms in which SCP may be consumed are continuing to evolve. Yeast SCP has been consumed for decades as a cell extract in the form of pastes which can be spread on bread, whereas the fungal SCP which is used in Quorn™ was deliberately developed as a product which could be formulated into chunks and slices which would more closely resemble meat. More recently developed products are often formulated as dry powders or flours, which are intended to be mixed with other ingredients to create products in which the individual components are not perceived. Such products are suitable for incorporation into protein bars and beverages such as smoothies, which are currently popular. Additionally, solid state fermentations continue to be developed which use microbes to upgrade the protein quality and palatability of low nutrient plant products or ingredients. These are not strictly speaking SCP, since both the microbe and the original substrate contribute to the final product, but they will also contribute to the protein supply of the future. Having a broad range of food products which incorporate SCP should encourage further expansion of the market.

## Author contributions

AR, SH, and MT contributed equally to the researching and writing of this article. MW provided information on algal and fungal SCP, contributed to writing the article and reviewed and edited the manuscript.

### Conflict of interest statement

The authors are employees of VTT Technical Research Centre of Finland Ltd. and declare that the research was conducted in the absence of any commercial or financial relationships that could be construed as a potential conflict of interest. MW received financial support from Marlow Foods in 1986-1989 and worked in projects supported by Marlow Foods from 1989 to 1994, but has had no ongoing collaboration with them. The other authors declare that the research was conducted in the absence of any commercial or financial relationships that could be construed as a potential conflict of interest.

## Abbreviations

DSP: Downstream processing
GMO: Genetically modified organism
GRAS: Generally recognised as safe
SCP: Single cell protein
QPS: Qualified presumption of safety of micro-organisms in food and feed applications.
